# 

**Published:** 1895-05

**Authors:** 


### CONTENTS.

ORIGINAL COMMUNICATIONS—

Report of Part of My Surgical Work for the Six Months Ending April 15th, with Remarks. By J. B. S. Holmes, M.D., Atlanta, Ga.......... 129

Mental Suggestion as an Aid in the Treatment of Morphinomania. By Sam'l H. Green, M.D., Oakdale, Ga.......... 140

Modern Obstetrical Practices. By C. D. Hurt, M.D., Atlanta, Ga.......... 144

A Report of Two Cases from Notes Found in My Record Book, with Remarks. By T. Emerson Mitchell, M.D., Columbus, Ga.......... 149

SOCIETY REPORTS—

Medical Association of Georgia.......... 153

EDITORIAL—

The Meeting in Savannah.......... 167

Hypnotism and Crime.......... 169

Examinations by the Regular Board of Medical Examiners, Atlanta, Ga., April 4, 1895.......... 170

SELECTIONS AND ABSTRACTS—

General Medicine and Therapeutics.......... 175

Eye, Ear, Nose, and Throat.......... 182

BOOK REVIEWS.......... 187

MEDICAL ITEMS.......... 190

ADVERTISERS' INDEX TO ADVERTISEMENTS.......... xii

MEDICAL ITEMS—Continued.......... ix, x, xi, xiv

